# Supplementary material for: Subtyping analysis reveals new variants and accelerated evolution of Clostridioides difficile toxin B
Source: Commun Biol. 2020 Jul 3;3:347. doi: 10.1038/s42003-020-1078-y (PMC7335066; doi:10.1038/s42003-020-1078-y)
Supplement: Supplementary file 1 — Supplementary information [file 42003_2020_1078_MOESM1_ESM.pdf]

# Supplementary Figures

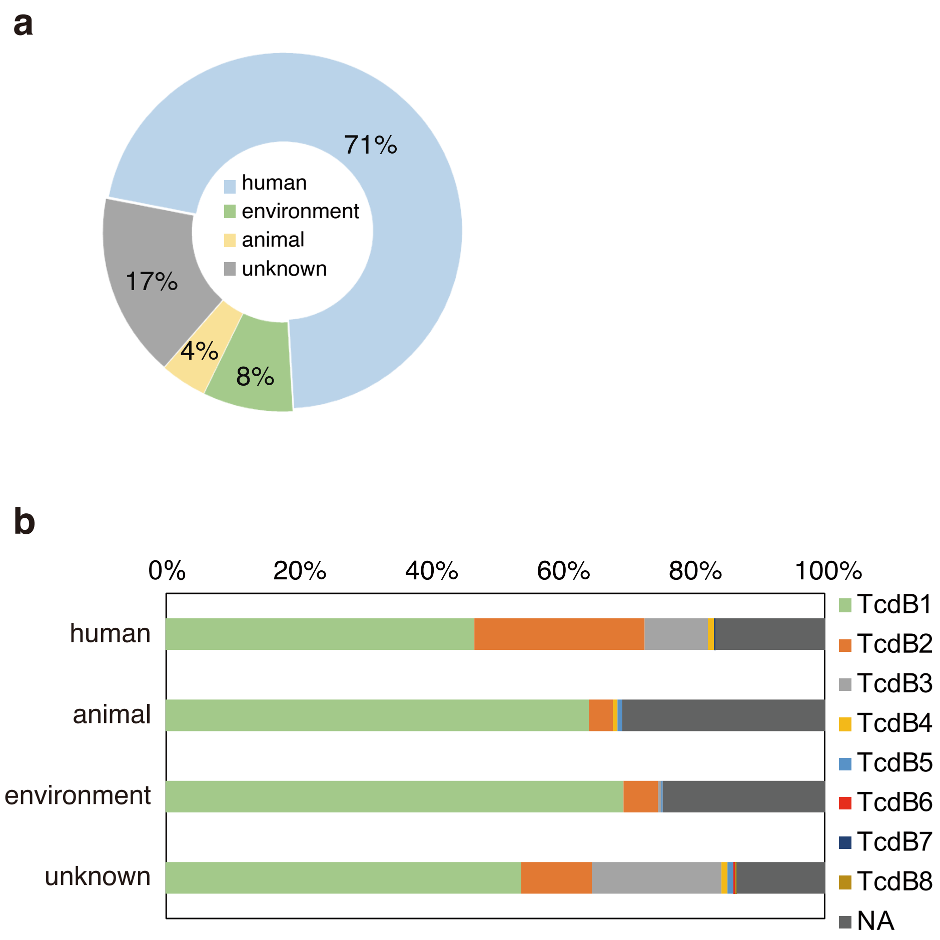


**Supplementary Figure 1**

**Breakdown of *C. difficile* genomes in this study.** (a) Breakdown of the *C. difficile* strains by different sources (human, animal, and environment). Approximately 71% of these isolates were originated from human samples, 8% were from environment like soil and water, 4% were from animal samples, and the rest (~17%) were from unknown sources. ‘Unknown’ means the absence of the source information. (b) The distribution of eight TcdB subtypes in different sources. ‘NA’ means that the sequence of TcdB was invalid or not identified in the genome.

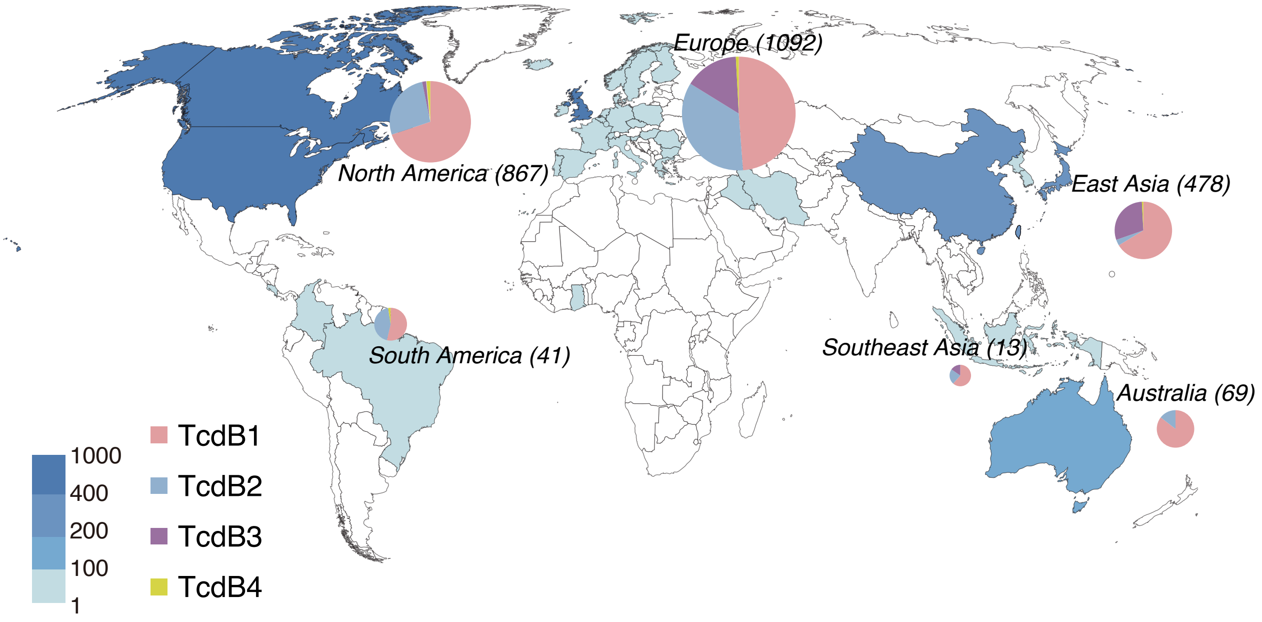


**Supplementary Figure 2**
**Geographical distribution of collected isolates.** The colors in countries represent the number of collected strains. The pie charts in the map showing the breakdown of genome-sequenced C. difficile strains in different geographical regions by TcdB subtypes 1-4. The world map was drawn by the module pygal.maps.world from the open source package pygal (http://www.pygal.org/en/stable/documentation/types/maps/pygal_maps_world.html).

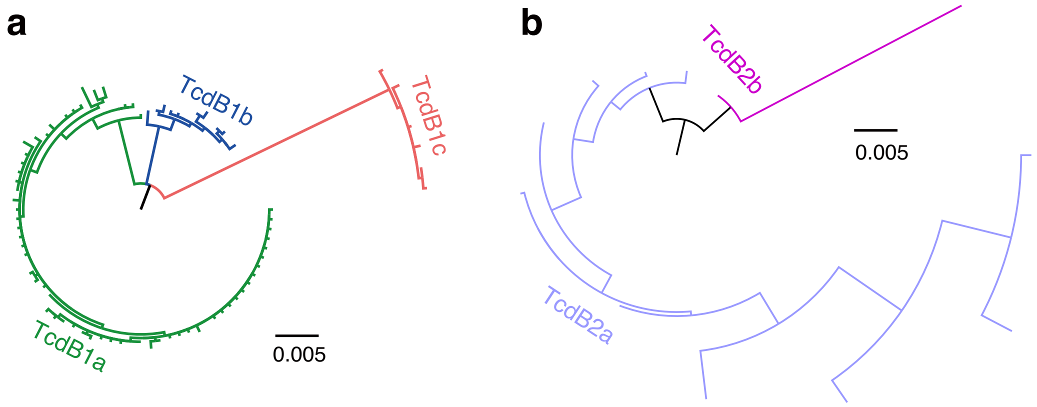


**Supplementary Figure 3**

**Maximum likelihood trees for TcdB1 and TcdB2.** (a) Three clades (TcdB1a, TcdB1b and TcdB1c) could be subdivided in TcdB1 tree. (b) Two clades (TcdB2a and TcdB2b) could be subdivided in TcdB2 tree.

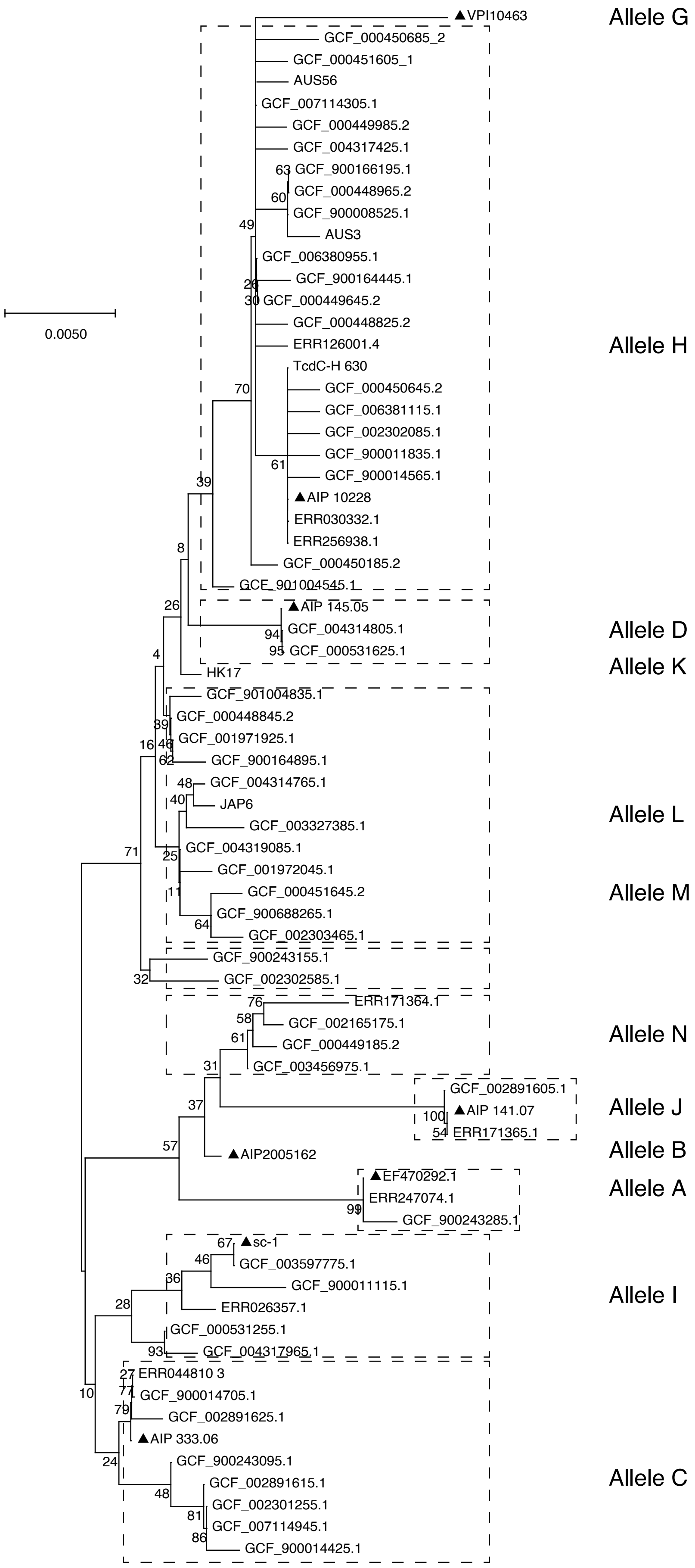


**Supplementary Figure 4**

**Dendrogram showing the genetic relationship of *C. difficile* isolates based on the nucleotide sequence of *tcdC*.** The sequences of the *tcdC* gene were retrieved from assembly genomes by local python scripts. After combing the same ones and removing low-quality sequences, the remaining 63 unique sequences and 8 sequences obtained from the previous study (marked with black triangles) were used to generate the tree with a bootstrap value of 1,000 replicates.

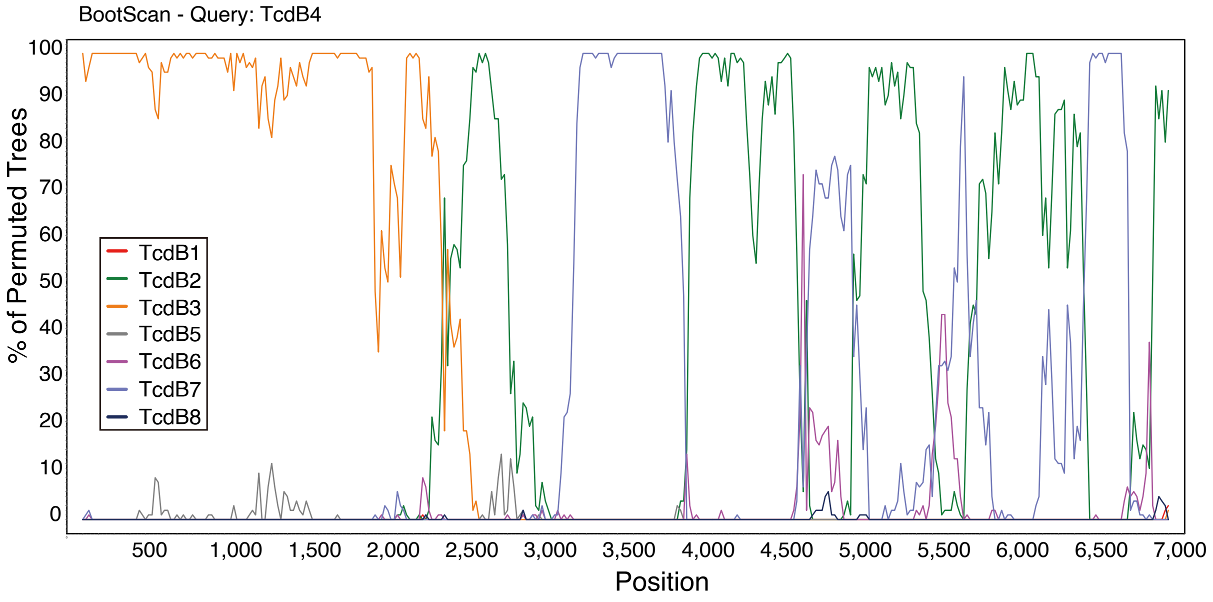


**Supplementary Figure 5**

**The bootscan analysis for the recombination origin of TcdB4.** TcdB4 was used as the query sequence and other TcdB subtypes were used as reference sequences.

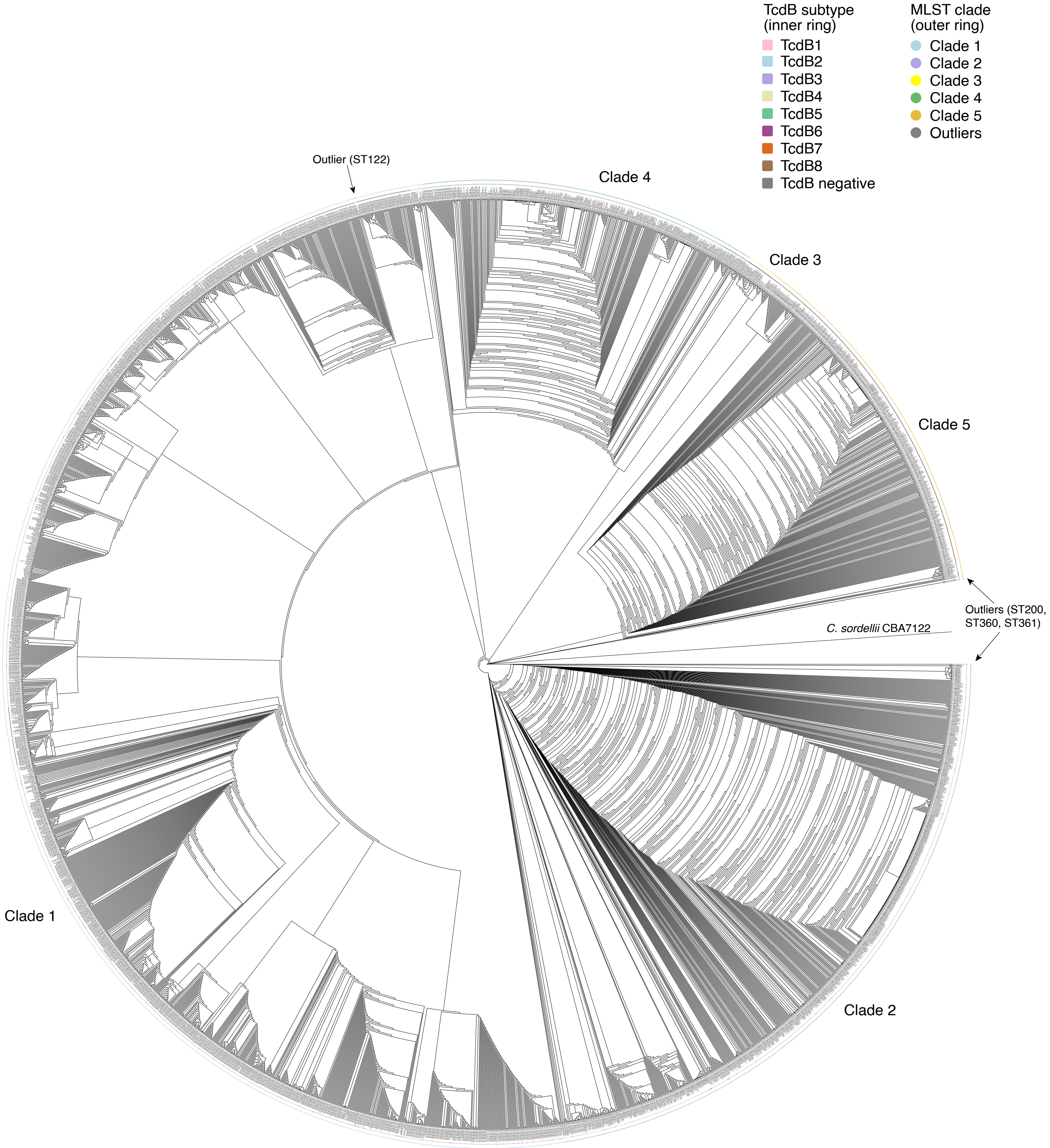


**Supplementary Figure 6**

**The distribution of TcdB subtypes in genome-wide SNP based phylogeny tree.** A parsimony tree was constructed with the core SNPs detected in the C. difficile genomes. C. sordellii CBA7122 was selected as outgroup. At the end of each branch, we marked two types of information, the inner ring represents the TcdB subtypes in each genome and the outer ring represents the clade information. Different colors denote different subtypes or clades. For TcdB1, we further marked TcdB1a, TcdB1b and TcdB1c in the figure. We used one diagonal line to further label TcdB1b and several vertical lines to label TcdB1c.

# Supplementary Tables

## Supplementary Table 1

### Maximum between-subtype amino acid differences among TcdB subtypes.

| Subtype | Numbers of Strains | Maximum Between-Subtype Differences (%) | | | | | | | |
|---|---|---|---|---|---|---|---|---|---|
| | | TcdB1 | TcdB2 | TcdB3 | TcdB4 | TcdB5 | TcdB6 | TcdB7 | TcdB8 |
| TcdB1 | n = 1652 | — | 9.05 | 9.63 | 15.00 | 8.66 | 11.67 | 12.09 | 8.24 |
| TcdB2 | n = 678 | | — | 12.84 | 10.10 | 13.18 | 7.90 | 7.61 | 9.76 |
| TcdB3 | n = 333 | | | — | 8.83 | 5.53 | 13.73 | 14.28 | 12.55 |
| TcdB4 | n = 29 | | | | — | 10.90 | 10.82 | 10.01 | 15.13 |
| TcdB5 | n = 7 | | | | | — | 14.41 | 14.53 | 12.25 |
| TcdB6 | n = 1 | | | | | | — | 6.09 | 11.33 |
| TcdB7 | n = 3 | | | | | | | — | 11.62 |
| TcdB8 | n = 1 | | | | | | | | — |

## Supplementary Table 2

## The geographical origins of different TcdB subtypes.

| Subtype | Country of origin | | | | | |
|---|---|---|---|---|---|---|
| | Europe | N. America | Asia | Oceania | S. America | Africa |
| TcdB1 | 533 | 606 | 334 | 59 | 22 | 1 |
| | United Kingdom(410), Netherlands(42), Belgium(24), Germany(21), Sweden(8), Ireland(6), France(4), Switzerland(3), Italy(2), Portugal(2), Greece(2), Hungary(2), Spain(2), Denmark(1), Norway(1), Bulgaria(1), Iceland(1), Slovenia(1) | United States(369), Canada(232), Costa Rica(5) | Japan(196), China(117), Iran(6), Singapore(5), Indonesia(3), North Korea(3), Iraq(2), Kuwait(1), South Korea(1) | Australia(59) | Colombia(22) | Ghana(1) |
| TcdB2 | 382 | 233 | 19 | 10 | 18 | 0 |
| | United Kingdom(332), Germany(14), Netherlands(13), Denmark(7), France(4), Poland(2), Luxembourg(2), Ireland(2), Switzerland(2), Belgium(2), Finland(1), Norway(1) | Canada(163), United States(68), Costa Rica(2) | Japan(8), Korea(5), Singapore(3), China(3) | Australia(10) | Colombia(18) | N.A. |
| TcdB3 | 168 | 15 | 143 | 0 | 0 | 0 |
| | Ireland(81), United Kingdom(59), Netherlands(16), Romania(2), Czech Republic(2), Poland(2), Greece(2), Bulgaria(2), Slovenia(1), Belgium(1) | United States(13), Canada(2) | China(69), Japan(33), North Korea(21), South Korea(18), Singapore(1), Indonesia(1) | N.A. | N.A. | N.A. |
| TcdB4 | 9 | 13 | 4 | 0 | 1 | 0 |
| | United Kingdom(7), Belgium(2) | United States(9), Canada(2), Costa Rica(2) | Japan(4) | N.A. | Brazil(1) | N.A. |
| TcdB5 | 0 | 0 | 0 | 7 | 0 | 0 |
| | N.A. | N.A. | N.A. | Australia(7) | N.A. | N.A. |
| TcdB6 | 0 | 1 | 0 | 0 | 0 | 0 |
| | N.A. | United States(1) | N.A. | N.A. | N.A. | N.A. |
| TcdB7 | 2 | 0 | 0 | 0 | 0 | 0 |
| | France(2) | N.A. | N.A. | N.A. | N.A. | N.A. |
| TcdB8 | 1 | 0 | 0 | 0 | 0 | 0 |
| | Spain(1) | N.A. | N.A. | N.A. | N.A. | N.A. |

## Supplementary Table 3

### The distribution of TcdB subtypes in different *C. difficile* clades.

| *C. difficile* clade | TcdB subtype | | | | | | | |
|---|---|---|---|---|---|---|---|---|
| | **TcdB1** | **TcdB2** | **TcdB3** | **TcdB4** | **TcdB5** | **TcdB6** | **TcdB7** | **TcdB8** |
| **Clade 1** | 1224 | 1 | 1 | 0 | 0 | 0 | 0 | 0 |
| **Clade 2** | 0 | 668 | 0 | 27 | 0 | 0 | 0 | 0 |
| **Clade 3** | 30 | 0 | 0 | 0 | 0 | 0 | 0 | 0 |
| **Clade 4** | 1 | 0 | 329 | 0 | 0 | 0 | 0 | 0 |
| **Clade 5** | 344 | 0 | 0 | 0 | 7 | 0 | 0 | 0 |
| **Outliers** | 0 | 0 | 0 | 0 | 0 | 1 | 3 | 1 |
