# Supplementary material for: Subtyping analysis reveals new variants and accelerated evolution of Clostridioides difficile toxin B
Source: Commun Biol. 2020 Jul 3;3:347. doi: 10.1038/s42003-020-1078-y (PMC7335066; doi:10.1038/s42003-020-1078-y)
Supplement: Supplementary file 2 — Description of additional supplementary files [file 42003_2020_1078_MOESM2_ESM.pdf]

Description of Additional Supplementary Files

File Name: **Supplementary Data 1**

Description: Source data underlying graphs and charts shown in figures and tables are provided in Supplementary Data 1.
